# Structural basis for high-affinity recognition of aflatoxin B1 by a DNA aptamer

**DOI:** 10.1093/nar/gkad541

**Published:** 2023-06-23

**Authors:** Guohua Xu, Chen Wang, Hao Yu, Yapiao Li, Qiang Zhao, Xin Zhou, Conggang Li, Maili Liu

**Affiliations:** Key Laboratory of Magnetic Resonance in Biological Systems, State Key Laboratory of Magnetic Resonance and Atomic and Molecular Physics, National Center for Magnetic Resonance in Wuhan, Wuhan National Laboratory for Optoelectronics, Wuhan Institute of Physics and Mathematics, Innovation Academy for Precision Measurement Science and Technology, Chinese Academy of Sciences, Wuhan 430071, P.R. China; Key Laboratory of Magnetic Resonance in Biological Systems, State Key Laboratory of Magnetic Resonance and Atomic and Molecular Physics, National Center for Magnetic Resonance in Wuhan, Wuhan National Laboratory for Optoelectronics, Wuhan Institute of Physics and Mathematics, Innovation Academy for Precision Measurement Science and Technology, Chinese Academy of Sciences, Wuhan 430071, P.R. China; Department of Chemistry, University of Chinese Academy of Sciences, Beijing 100049, P.R. China; State Key Laboratory of Environmental Chemistry and Ecotoxicology, Research Center for Eco-Environmental Sciences, Chinese Academy of Sciences, Beijing 100085, P.R. China; Department of Chemistry, University of Chinese Academy of Sciences, Beijing 100049, P.R. China; State Key Laboratory of Environmental Chemistry and Ecotoxicology, Research Center for Eco-Environmental Sciences, Chinese Academy of Sciences, Beijing 100085, P.R. China; Department of Chemistry, University of Chinese Academy of Sciences, Beijing 100049, P.R. China; State Key Laboratory of Environmental Chemistry and Ecotoxicology, Research Center for Eco-Environmental Sciences, Chinese Academy of Sciences, Beijing 100085, P.R. China; School of Environment, Hangzhou Institute for Advanced Study, University of Chinese Academy of Sciences, Hangzhou 310024, P.R. China; Department of Chemistry, University of Chinese Academy of Sciences, Beijing 100049, P.R. China; Key Laboratory of Magnetic Resonance in Biological Systems, State Key Laboratory of Magnetic Resonance and Atomic and Molecular Physics, National Center for Magnetic Resonance in Wuhan, Wuhan National Laboratory for Optoelectronics, Wuhan Institute of Physics and Mathematics, Innovation Academy for Precision Measurement Science and Technology, Chinese Academy of Sciences, Wuhan 430071, P.R. China; Key Laboratory of Magnetic Resonance in Biological Systems, State Key Laboratory of Magnetic Resonance and Atomic and Molecular Physics, National Center for Magnetic Resonance in Wuhan, Wuhan National Laboratory for Optoelectronics, Wuhan Institute of Physics and Mathematics, Innovation Academy for Precision Measurement Science and Technology, Chinese Academy of Sciences, Wuhan 430071, P.R. China; Key Laboratory of Magnetic Resonance in Biological Systems, State Key Laboratory of Magnetic Resonance and Atomic and Molecular Physics, National Center for Magnetic Resonance in Wuhan, Wuhan National Laboratory for Optoelectronics, Wuhan Institute of Physics and Mathematics, Innovation Academy for Precision Measurement Science and Technology, Chinese Academy of Sciences, Wuhan 430071, P.R. China

## Abstract

The 26-mer DNA aptamer (AF26) that specifically binds aflatoxin B1 (AFB1) with nM-level high affinity is rare among hundreds of aptamers for small molecules. Despite its predicted stem–loop structure, the molecular basis of its high-affinity recognition of AFB1 remains unknown. Here, we present the first high-resolution nuclear magnetic resonance structure of AFB1–AF26 aptamer complex in solution. AFB1 binds to the 16-residue loop region of the aptamer, inducing it to fold into a compact structure through the assembly of two bulges and one hairpin structure. AFB1 is tightly enclosed within a cavity formed by the bulges and hairpin, held in a place between the G·C base pair, G·G·C triple and multiple T bases, mainly through strong π–π stacking, hydrophobic and donor atom–π interactions, respectively. We further revealed the mechanism of the aptamer in recognizing AFB1 and its analogue AFG1 with only one-atom difference and introduced a single base mutation at the binding site of the aptamer to increase the discrimination between AFB1 and AFG1 based on the structural insights. This research provides an important structural basis for understanding high-affinity recognition of the aptamer, and for further aptamer engineering, modification and applications.

## Graphical Abstract

**Graphical Abstract:**
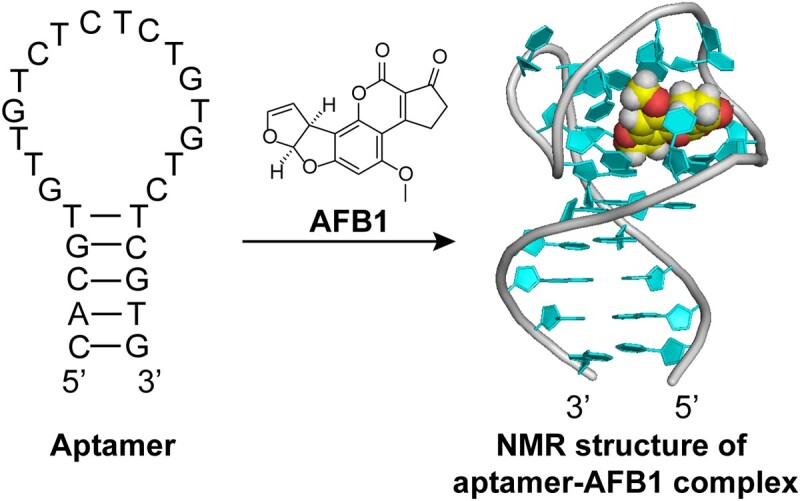
Aflatoxin B1 binds to the 16-residue loop region of the aptamer, inducing it to fold into a compact structure.

## INTRODUCTION

Aptamers are single-stranded DNA or RNA oligonucleotides that are easily available, yet capable of binding to their targets with high affinity and specificity, making them have diverse and increasing applications in biosensors, disease diagnosis, therapeutics, etc. (1–11). Aptamers are selected for small-molecule targets such as drugs, toxins, biomarkers and pollutants, and have become powerful tools for small-molecule analysis and detection (5,12–17). Compared to the aptamers for proteins, aptamers for small molecules usually have dissociation constant (*K*_d_) at μM or sub-μM levels as the small molecules have fewer functional groups (15,17–21). However, a few aptamers for small molecules exhibit strong affinity, with *K*_d_ values at nM levels. Among them, the aptamer against aflatoxin B1 (AFB1) has attracted intense and increasing attention due to its high affinity and selectivity (22–25).

**Figure 1.**
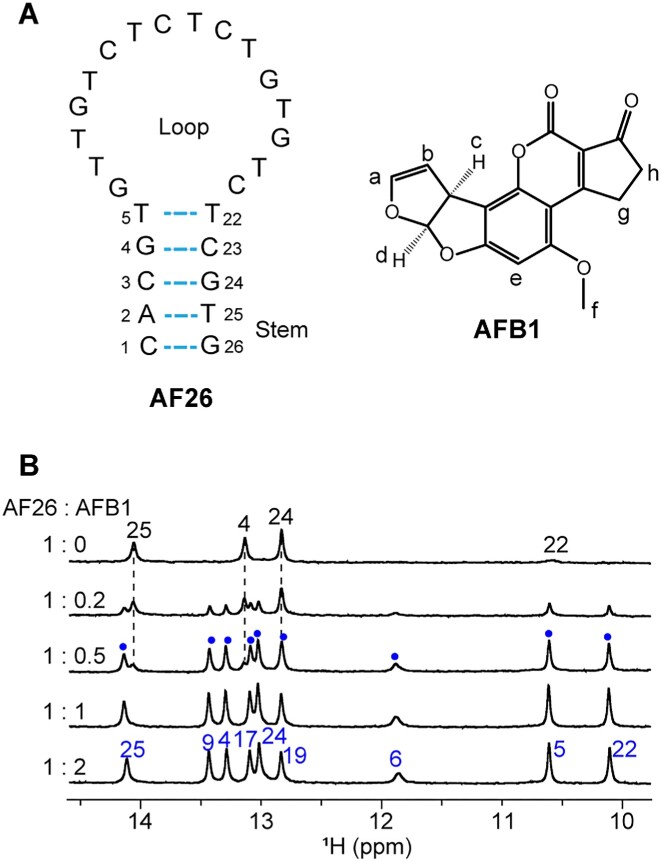
^1^H nuclear magnetic resonance (NMR) spectra of aptamer AF26 upon binding with AFB1. (**A**) Sequence of aptamer AF26 and chemical structure of AFB1. (**B**) Imino regions of ^1^H NMR spectra of aptamer AF26 titrated with AFB1 at 278 K. The signal assignments of imino protons from free and bound aptamers are marked in black and blue fonts, respectively. The peaks arising from the complex at the 1:0.5 ratio of AF26 to AFB1 are marked with blue points.

Aflatoxins, secondary metabolites of *Aspergillus flavus* and *A. parasiticus*, are a type of food-contaminating small-molecule mycotoxin. Among aflatoxins, AFB1 (Figure 1) is considered the most toxic, being a potent carcinogen for humans and animals (26–28). Due to its toxicity and frequent contamination in various foods, the detection of AFB1 is important for food safety, environment monitoring and risk assessment (28,29). A selected 50-mer DNA aptamer (AF50, 5′-GTTGGGCACGTGTTGTCTCTCTGTGTCTCGTGCCCTTCGCTAGGCCCACA-3′) is able to bind AFB1 with a *K*_d_ of tens of nM and exhibit good selectivity against other mycotoxins (30). As a result, this aptamer and its truncated sequences have been used for AFB1 detection with various methods (e.g. fluorescence, electrochemistry, etc.) in environmental monitoring, food safety and quality control (23,31–34). A50 is predicted to contain a simple stem–loop structure that is demonstrated to be essential for high-affinity binding to AFB1 (30,35). The 26-mer aptamer sequence AF26 with 24 nucleotides truncated from both ends of A50 (AF26, 5′-CACGTGTTGTCTCTCTGTGTCTCGTG-3′), containing the key stem–loop structure, has been found to be the shortest sequence capable of strongly binding to AFB1 with affinity at nM levels (Supplementary Table S1) (35). In addition, a variety of mutations show that the aptamer binding affinity highly depends on the sequence of loop (35), implying that AFB1 may bind at the loop region. However, the detailed molecular mechanism of the rare, specific, high-affinity recognition of AFB1 by its aptamer remains unclear.

Herein, we determined the high-resolution solution structure of AFB1–AF26 complex by NMR spectroscopy, providing a deeper insight into the molecular mechanism of tight and specific binding between aptamer and AFB1. This study will provide structural foundation for the aptamer engineering and modification for broad applications with rational designs, and it is also helpful for selection of high-affinity ligands of small molecules.

## MATERIALS AND METHODS

### Sample preparation

Aflatoxins B1 and G1 were purchased from Shanghai Yuanye Bio-Technology Co., Ltd and J&K Scientific (Beijing, China), respectively, and the aflatoxins were dissolved in dimethyl sulfoxide-d_6_ (DMSO) as stock solution for further use. Tris (D11, 98%) and sodium 2,2-dimethyl-2-silapentane-5-sulfonate (DSS) were from Cambridge Isotope Laboratories. DNA oligos were synthesized by Sangon Biotechnology Co., Ltd (Shanghai, China). NMR samples were prepared by dissolving the lyophilized DNA powder in either D_2_O or 90% H_2_O/10% D_2_O containing 10 mM Tris (1D ^1^H NMR spectra) or Tris-d_11_ (2D NMR spectra), 50 mM NaCl and 50 mM MgCl_2_ (pH 6.5–7.5). For 1D ^1^H NMR spectra, low-concentration DNA samples (50–100 μM) were used. For 2D NMR study, DNA samples with higher concentration (1–3 mM) were used, and 1–2 equivalents of AFB1 were added.

### NMR spectroscopy

NMR spectra were recorded on Bruker 600, 700 or 850 MHz NMR spectrometers equipped with a CyroProbe, respectively. The W5 water suppression was used for 1D ^1^H NMR spectra. 2D ^1^H–^1^H (Total Correlation Spectroscopy) TOCSY (120 ms mixing time) and (Double-Quantum Filtered Correlation Spectroscopy) DQF-COSY spectra were acquired in D_2_O at 278 and 298 K. 2D ^1^H–^1^H (Nuclear Overhauser Effect Spectroscopy) NOESY spectra were collected in D_2_O and 90% H_2_O/10% D_2_O, respectively, with mixing time of 120 and 300 ms, using W5 or jump-and-return water suppression at 278 and 298 K. ^1^H chemical shifts were referenced to DSS at 0 ppm. 2D ^15^N–^1^H Selective Optimized Flip Angle Short Transient-Technique coupled to Heteronuclear Multiple Quantum Correlation (SOFAST-HMQC) spectrum was acquired with 10 K scans at 278 K. 2D ^13^C–^1^H *J*-resolved heteronuclear multi-bond correlation (HMBC) spectrum was collected with 16 K scans at 278 K. 2D ^31^P–^1^H COSY spectra were recorded in D_2_O at 298 K. ^31^P chemical shifts were referenced to an external standard of 85% H_3_PO_4_. The NMR spectra were processed and analyzed using Bruker TopSpin and Sparky software (36).

### Structure calculation

The structures of AF26–AFB1 complex were calculated following the standard protocol using Xplor-NIH 2.47 (37–39), as described previously (16,40). Hydrogen bonding, intramolecular and intermolecular NOE distance restraints, and dihedral angle restraints were used in calculation. NOE distance restraints were mainly based on NOESY spectra with mixing time of 120 ms. The distances were loosely classified into three categories: strong (1.8–2.5 Å), medium (1.8–3.5 Å) and weak (1.8–5.5 Å). Sugar puckering conformations were restricted based on the intensities of cross-peaks in COSY spectra. The glycosidic dihedral angles (*χ*) were restricted based on intraresidue H1′–H6/8 cross-peak intensities. The *β* and *ϵ* angles were restrained based on the *J*-coupling constants of P(*n*)–H5′/H5″(*n*) and H3′(*n* − 1)–P(*n*) obtained from ^31^P–^1^H COSY, respectively. AFB1 parameter files were obtained from PRODRG (41). A total of 200 structures were obtained and the 10 lowest-energy structures were chosen for further analysis. The structures of AF26–AFG1 complex were calculated by the identical method to the AF26–AFB1 complex. Structures were analyzed and displayed using PyMOL Molecular Graphics System, version 0.99rc6 (Schrödinger, LLC).

### Isothermal titration calorimetry analysis

The aptamers and AFB1 solutions were prepared in the buffer of 10 mM Tris–HCl (pH 7.5), 50 mM MgCl_2_, 50 mM NaCl, 2% DMSO and 0.1% Tween 20. Characterization of affinity binding between aptamers and AFB1 was conducted at 298 K by using a MicroCal PEAQ-ITC microcalorimeter (Malvern). During isothermal titration calorimetry (ITC) analysis, 5 μM aptamer in binding buffer in sample cell was gradually titrated by AFB1 solution (50 μM) in injection syringe, with a reference power at 10.00 μcal/s and a stirring speed of the syringe at 750 rpm. After an initial 60 s equilibrium step, first 0.4 μl injection for 0.8 s was conducted followed by 19 successive 2.0 μl injections (each injection was kept at 4 s) every 100 s. Dissociation constants (*K*_d_) of aptamers were obtained by a one-set binding model fitting with the packaged MicroCal PEAQ-ITC analysis software.

### Surface plasmon resonance assay

Surface plasmon resonance (SPR) analyses of binding between AF26 and target were performed with Biacore T200 (GE Healthcare) at 25°C. An SPR SA sensor chip with four flow cells was used. Flow cell 1 was used as a reference cell, while flow cell 2 was used as an experimental cell. Running buffer contained 10 mM Tris–HCl (pH 7.5), 50 mM MgCl_2_, 50 mM NaCl, 0.1% Tween 20 and 0.2% DMSO. Biotinylated AF26 was conjugated on the sensor chip by injecting the aptamer (25 nM) in running buffer into flow cell 2 with a flow rate of 2 μl/min for 400 s. The biotinylated aptamer had one biotin labeled at the 5′ terminal of aptamer with a triethylene glycol spacer. The biotinylated AF26 was attached to the surface of the SA sensor chip with an amount bound of 643 RU (response unit). To characterize the affinity binding between aptamer and AFB1, AFB1 at different concentrations in running buffer was injected at a flow rate of 30 μl/min for 120 s. The SPR signal was recorded in real time. After each injection, further running for 250 s was applied to dissociate the bound AFB1, allowing the signal back to the baseline. The data were processed by Biacore T200 Evaluation Software, version 2.0.

## RESULTS

### NMR titration of AF26 with AFB1

First, we performed ^1^H NMR titration of AF26 with AFB1 to investigate the aptamer–AFB1 binding. In the absence of AFB1, free AF26 did not give rise to any signal in the imino proton region at 298 K, but displayed signals at 278 K (Figure 1, and Supplementary Figures S1 and S2). These signals were assigned to the imino protons of the stem of aptamer (the assignments are described later) (Supplementary Figure S2 and Supplementary Table S2), suggesting that free AF26 forms a stem–loop structure as predicted (Figure 1). The phenomenon that the imino protons of the stem yield signals at lower temperature, but not at room temperature, indicates that the stem’s stability is related to temperature, and low temperature stabilizes the stem.

As AFB1 was titrated into AF26 at 278 K, the peaks from the free AF26 gradually decreased and meanwhile a new set of well-resolved imino protons for the AFB1–AF26 complex appeared (Figure 1). This result suggests that AFB1 binding induced AF26 to fold into a new, well-defined structure and the binding of AF26 to AFB1 occurred in a slow exchange on the NMR timescale, allowing the free and bound states of AF26 to be observed simultaneously. When 1 equivalent of AFB1 was added, only one set of peaks from the complex were observed, and the peaks did not further change upon the addition of 2 equivalents of AFB1, indicative of the formation of 1:1 complex between AF26 and AFB1, which is consistent with the determined stoichiometry by ITC analysis (Supplementary Figure S3). Furthermore, in contrast to fewer signals of imino protons for the free AF26 aptamer, the eight narrow and one broader imino protons were observed for the bound AF26 aptamer (278 K), indicating that a structured loop may form on complex formation. The high-quality proton NMR spectra of the AFB1–AF26 complex at 278 and 298 K also indicate that it is suitable to resolve the NMR structure of the complex.

### Resonance assignments of free AF26 aptamer and AFB1–AF26 complex

The NMR signals of the imino protons of free AF26 are sensitive to temperature. The ^1^H spectrum did not yield any signal in the imino proton region at 298 K, but displayed signals at 278 K (Supplementary Figures S1 and S2). These signals were assigned based on the G-to-I and T-to-dU substitutions and NOESY spectra (Supplementary Figure S2). Due to fast exchange rate of imino protons, T5, T22 and G26 can only be clearly observed at acidic pH (<7.0). These results of assignments indicate that the stem of free aptamer forms through canonical G·C and A·T Watson–Crick base pairing and T5·T22 mismatch between C1–A2–C3–G4–T5 and T22–C23–G24–T25–G26 (Figure 1).

**Figure 2.**
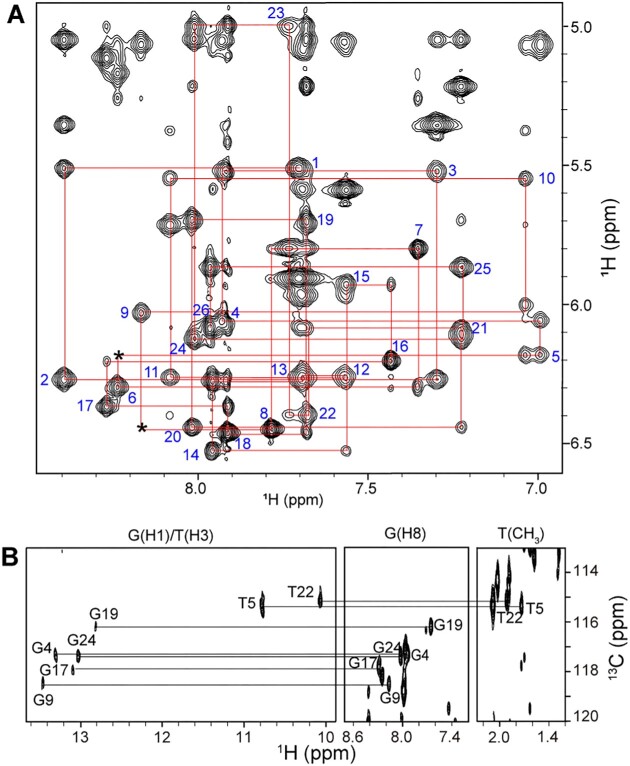
Aptamer resonance assignments in the AFB1–AF26 complex. (**A**) NOESY spectrum (mixing time, 300 ms) of complex in D_2_O buffer at 278 K, showing the H8/6–H1′ sequential connectivities. Intraresidue H8/6–H1′ cross-peaks are labeled with residue numbers. Missing connectivities are marked with asterisks. (**B**) HMBC spectrum of the complex at natural abundance at 278 K, showing G(H1)/T(H3) and G(H8)/T(CH_3_) proton assignments by through-bond correlativities via ^13^C5.

To determine the structure of the complex, we acquired a series of 2D NMR spectra, including TOCSY, COSY, NOESY and HMBC. Groups of protons in specific sugars or bases can be identified by their scalar couplings in COSY and TOCSY spectra. The AF26 aptamer is a T- and C-rich sequence. The H5–H6 and CH_3_–H6 cross-peaks from C and T bases can be unambiguously observed in TOCSY and COSY spectra, which were used to help assign the base types of H8/6 in NOESY spectra. The well-resolved NOESY spectra allow us to trace the H8/6–H1′ and H8/6–H2′/H2″ sequential connectivities (the NOESY spectra at different temperatures can help distinguish the overlapped signals) (Figure 2). The scalar connectivities of H2′/2″–H3′–H4′–H5′/5″ in TOCSY spectra allow for the identification of the spin systems. The sequential H3′(*n* − 1)–P(*n* − 1)–H4′/H5′/5″(*n*) ^1^H–^31^P correlations were also used to aid the sequential and H3′, H4′ and H5′/5″ proton assignments. Some protons yield unusual resonances. The H4′ of T7, the H4′ of C15 and H5′/5″ of T16, and the H5′/5″ of T22 indicated the unusual upfield-shifted resonances due to the ring current effect of G9, G17 and C11 bases, respectively. The methyl protons of T10 also showed unusual upfield shift due to the ring current effect of both G6 and T5. Additionally, the H4′ of G6 indicated the unusual downfield-shifted resonances due to deshielding effect caused by adjacent electronegative group (Supplementary Table S3). Most of the imino protons were assigned by the heteronuclear ^13^C–^1^H HMBC spectrum of the complex at natural abundance, which connects the imino protons to the guanosine H8 and thymidine CH_3_ protons via ^13^C5 (Figure 2 and Supplementary Table S3). The remaining imino protons such as G6, T10 and T25, which were not observed in the ^13^C–^1^H HMBC spectrum, were identified by the G-to-I substitution (Supplementary Figure S4 and Supplementary Table S2) and by the NOEs of the imino protons of thymidine bases to its own methyl protons. The NMR signals of imino protons of T10 and T7 are sensitive to pH values, and can only be observed at pH ≤7 (Supplementary Figure S5). After the proton signals of the aptamer were fully identified, the proton signals of bound AFB1 can be easily assigned by analyzing the remaining unassigned peaks (Supplementary Table S4).

### Solution structure of the AFB1–AF26 aptamer complex and binding pocket

**Figure 3.**
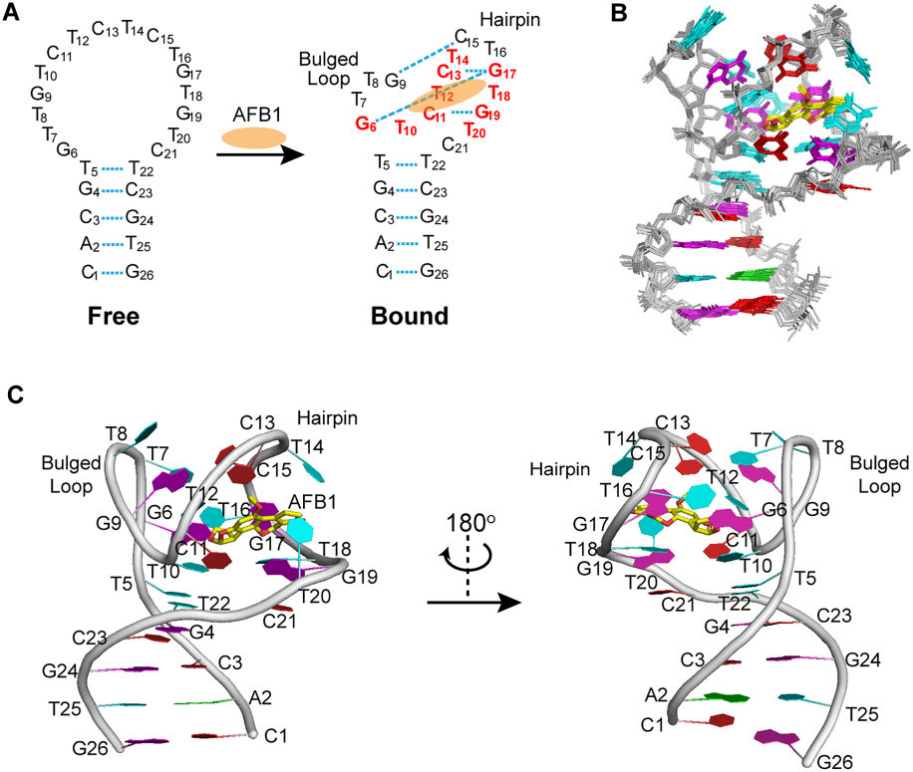
The overall structure of the AFB1–AF26 complex. (**A**) Secondary structure of free and bound AF26 aptamers. AFB1 is shown as orange ellipse. The base pairs are shown in cyan dash lines. The residues forming the AFB1 binding site are colored red. (**B**) Ten superimposed refined structures. (**C**) Cartoon view of a representative refined structure. Guanines are colored magenta; cytosines, red; adenines, green; thymines, cyan; backbone, gray; AFB1, yellow for carbon and red for oxygen atoms.

In the absence of AFB1, the free aptamer forms a stem–loop structure (Figure 3). Upon AFB1 binding, we clearly identified that the 16-residue loop undergoes an adaptive conformational transition from unstructured to structured, based on the unambiguous assignment of AF26 and AFB1 proton resonances. This is reflected in the stable formation of the G9·C15 and G19·C11 Watson–Crick pairs and the G6·G17·C13 triple, which are supported by the characteristic NOE peaks, such as the strong NOEs between the imino proton of G base and the amino protons of C base for G·C pairing (Figure 3A and Supplementary Figure S6). In addition, the intermolecular NOEs between the protons of AFB1 and G6, T10, C11, T12, C13, T14, G19 and T20 of the aptamer AF26 indicate that AFB1 specially binds in the loop region of AF26 (Figure 3 and Supplementary Table S5).

The high-resolution NMR structure of the complex was calculated using NOE-derived distance, hydrogen bonds and dihedral angle restraints (Figure 3, and Supplementary Tables S5 and S6). A total of 395 NOE distance restraints, including 49 intermolecular restraints between AF26 and AFB1, were used for the calculation, which clearly defined the position of AFB1 in complex and the overall complex structure. Ten lowest-energy refined structures of the complex are presented in Figure 3B. These structures are well converged and have an overall pair root-mean-square deviation value of 0.54 ± 0.17 Å for all heavy atoms of the complex (Supplementary Table S6).

**Figure 4.**
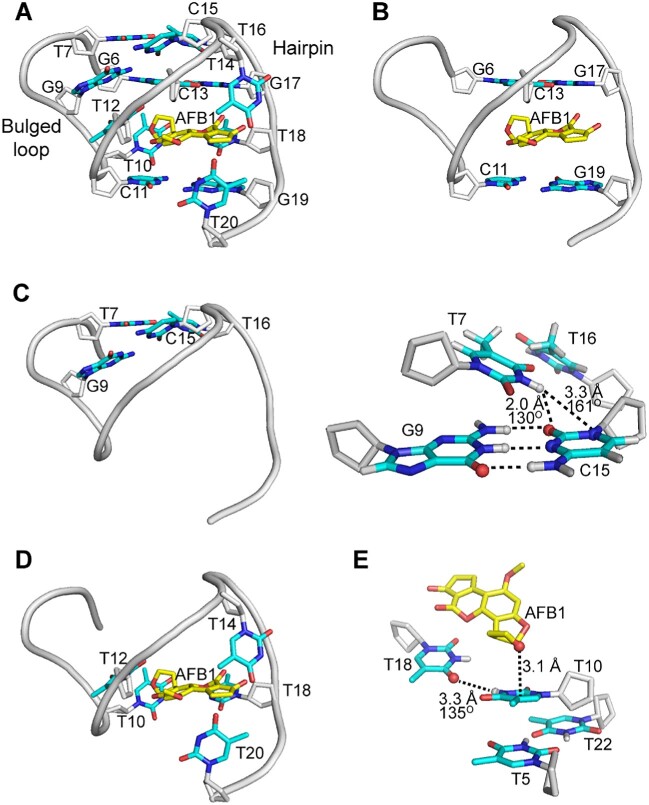
A detailed view of AFB1 binding site in a representative refined AFB1–AF26 complex structure. (**A**) An overall view of AFB1 binding pocket. (**B**) Side view showing AFB1 interacting through strong π–π stacking with the G19·C11 base pair and the G6·G17·C13 triple. (**C**) The kissing loop interaction between the hairpin loop and the bulged G6–T10 loop. (**D**) Side view showing that AFB1 is wrapped around by T bases, interacting through hydrophobic effects. (**E**) The donor atom–π interaction between T10 and AFB1, the hydrogen bonding interaction between T10 and T18, and the stacking interaction between T10 and T5·T22 mismatch pair. AFB1 was colored yellow for carbon and red for oxygen atoms. For AF26 aptamer, the sugar rings were colored gray, and the bases are depicted in red for oxygen, blue for nitrogen, cyan for carbon and gray for hydrogen atoms.

The high-resolution structure shows that the AFB1 binding pocket is formed by one C11–G19 hairpin and two bulges of five-residue G6–T10 and two-residue T20–C21. The hairpin structure folds toward the bulged G6–T10 loop, which, together with the T20–C21 loop, creates a compact structure for binding AFB1. The bound AFB1 molecule is tightly and almost completely encapsulated within the binding pocket (Figures 3 and 4).

The bulged G6–T10 loop and the C11–G19 hairpin are particularly significant components of the complex structure, providing the skeletal framework of the binding pocket (Figure 4A). The G19·C11 base pair and the G6·G17·C13 triple, spanning the hairpin and bulged G6–T10 loop, sandwich AFB1 through a strong π–π stacking interaction (Figure 4B). Further, the hairpin loop T14–C15–T16 and the bulged G6–T10 loop are held together by a kissing loop interaction, which drives the tip of hairpin loop to fold toward the bulged loop through the Watson–Crick pairing of G9 in the bulge with C15 in the hairpin loop (Figure 4C). Additionally, the H3 proton of T7 forms a hydrogen bond with C15 base, and the methyl group of T7 interacts with the methyl group of T16 through the hydrophobic effect. These kissing interactions help to stitch the bugled loop and the hairpin, thereby shaping the entire binding pocket for AFB1. The significance of the kissing interactions was confirmed by the G9 mutation, as ITC results show that any replacement of G9 base with A, T, C or inosine (I), which destroys G9·C15 pair to varying extents, causes a complete loss of aptamer affinity at 298 K (Supplementary Tables S2 and S7). The ^1^H NMR spectra of AF26–G9I also show that the signals of the bound aptamer, such as the imino proton signals of T5 and T22, cannot be observed at 298 K (though they can be observed at 278 K, likely because low temperature enhances structure stability and facilitates the binding) (Supplementary Figure S3). Moreover, the completely lost or significantly reduced affinity caused by the replacement of T7 with A7 (AF26–T7A, no binding), T16 with A16 (AF26–T16A, *K*_d_ = 2.84 ± 2.19 μM), indicates the importance of the specific loop–loop interaction between the bulge and the hairpin. The substitutions of T7 or T16 with dU cause reduced affinity (Supplementary Tables S2 and S7), confirming the existence of the interaction between the methyl groups.

The binding pocket has another unique feature where five unpaired T bases (T10, T12, T14, T18 and T20) wrap around AFB1 like a belt (Figure 4D). The T10 base forms a donor atom–π interaction (42,43) with the oxygen atom of the outer dihydrofuran ring of AFB1 and also interacts with the ring of AFB1 through methyl hydrophobic effect. The stacking and hydrophobic interactions between T10 and the T5·T22 mismatch pair, and the hydrogen bond formation between H3 of T10 and O4 of T18 base, provide an important support for T10 to interact with AFB1 in a favorable orientation (Figure 4E). The T12 base is inserted below the G9 base by stacking interaction, which stabilizes the kissing interaction between the hairpin loop and the bulged G6–T10 loop. The sugar ring of T12, along with the sugar ring of C13, also directly interacts with the methyl group of AFB1 through hydrophobic effect (Figure 4A). The looped out T14 and T20 bases interact with the two CH_2_ groups of cyclopentanone of AFB1 from above and below, respectively, through the hydrophobic effects of their methyl groups. The hydrophobic interactions between the methyl group of T14 and the sugar ring of C13, and between the methyl group of T20 and the sugar ring of G19, help to stabilize the orientation of the two looped out T bases (Figure 4A). The importance of these five surrounded T bases for binding can be reflected to a certain extent by the completely lost or significantly reduced affinity caused by the substitution of T with A, respectively (AF26-T10A and AF26-T12A, no binding; AF26-T14A, *K*_d_ = 1.53 ± 0.511 μM; AF26-T18A, *K*_d_ = 2.00 ± 1.50 μM; AF26-T20A, *K*_d_ = 3.28 ± 3.78 μM). The hydrophobic contribution of methyl groups of these T bases to binding can be confirmed by the substitution of T with dU, respectively. The ITC results indicate that the substitution of T10 with dU results in the 10-fold reduced affinity, showing the importance of the methyl group of the T10 base for binding (Supplementary Tables S2 and S7).

In summary, the wrapping of five T bases from the sides, along with the sandwich packing of the G·C base pair and G·G·C base triple from above and below, almost completely encloses AFB1 within the aptamer binding pocket (Supplementary Figure S7). This unique structural feature is likely to provide substantial contributions to the observed nM binding affinity of aptamer. The SPR study shows that the aptamer–AFB1 binding has a low dissociation rate constant (*K*_off_) of ∼0.046 s^−1^ (Supplementary Figure S8), confirming that the wrapped AFB1 by the aptamer is tightly bound, which greatly enhances the binding affinity.

We have noted that many aptamers adopt stem–loop structures when binding small-molecule ligands, such as aptamers that bind to tobramycin/neomycin, l-argininamide and calicheamicin (12,44–48). However, these aptamers utilize different binding modes from the AF26. They form ligand-binding pockets mainly by hairpin loop folding toward the stem (e.g. tobramycin/neomycin-binding aptamers), or binding the ligand in the stem (e.g. calicheamicin-binding aptamers). In contrast, AF26 creates a new and unique aptamer-binding motif by generating a binding pocket consisting of the bulged loop and the hairpin, which tightly encapsulates the AFB1. The AFB1 induced the unstructured loop to fold into such a structure of unprecedented complexity, making it challenging for current computation simulations to generate an accurate structure model (49–51).

### Structural insights into AF26 discrimination between AFB1 and AFG1

**Figure 5.**
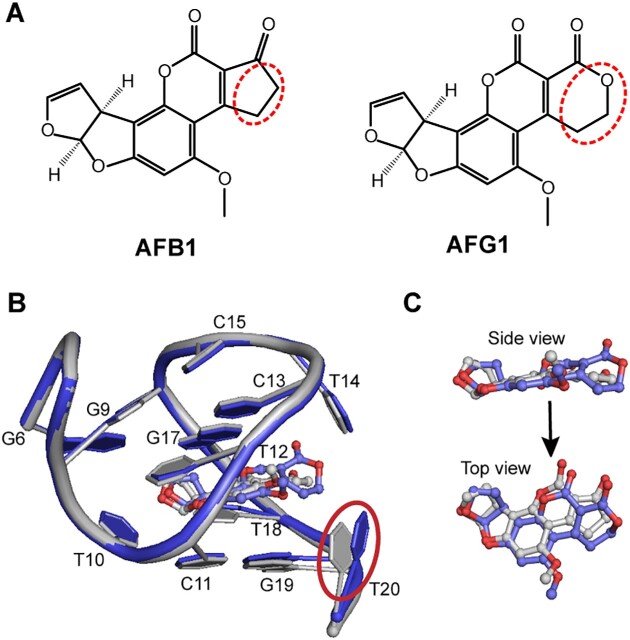
The comparison of the mean structures of AFB1–AF26 and AFG1–AF26 complexes. (**A**) Chemical structures of AFB1 and AFG1. The difference of the two structures is marked with red dash circles. (**B**) The structural comparison of binding pockets. The obvious difference between the binding pockets of the two complexes is marked with red solid circle. (**C**) The comparison of bound AFB1 and AFG1 in complexes. AFB1 was colored gray for carbon and red for oxygen atoms; AFG1, blue for carbon and red for oxygen atoms. The AF26 aptamers bound to AFB1 and AFG1 were colored gray and blue, respectively.

AFG1 is an analogue of AFB1, which differs by only one atom. The cyclopentanone in AFB1 is replaced with dihydropyran-2-one in AFG1, with an addition of one oxygen atom (Figure 5A). ITC results show that AF26 aptamer has an ∼5-fold reduced *K*_d_ for AFG1. We speculated that the change from a five-membered ring to a six-membered ring might result in the steric effect that affects the affinity of AF26 to AFG1. To understand the details of discriminatory recognition, we acquired a series of NMR spectra and solved the high-resolution solution structure of AFG1–AF26 complex (Supplementary Tables S4 and S5). The NOESY spectra show that the signals of many protons of the aptamer shift compared to the AFB1–AF26 complex (Supplementary Figure S9). However, the intramolecular and especially intermolecular NOEs do not significantly change (Supplementary Table S6), suggesting that the difference between the structures of the two complexes is subtle. The overlap of high-resolution structures of the AFG1–AF26 and AFB1–AF26 complexes indicates that, as predicted, the most noticeable difference is the position of the T20 base (Figure 5B). In the AFG1–AF26 complex, the T20 base right next to the dihydropyran-2-one is flipped outward due to steric effect. Furthermore, the plane of AFG1 molecule is not as even as AFB1, reducing the stacking interaction between AFG1 and the aptamer (Figure 5C). These two factors may be the main reasons for the difference in affinity between AFB1 and AFG1 for AF26.

Based on the high-resolution structure of AFG1–AF26 and AFB1–AF26 complexes, we attempted to improve the aptamer’s ability to differentiate AFB1 and AFG1 by modifying or mutating the T20 base (Supplementary Tables S2 and S8). We replaced T20 with dU (AF26-T20-dU), which changes the methyl group in the T base into a hydrogen atom, and ITC results indicate that AF26-T20-dU exhibits ∼1.6-fold and ∼2.5-fold reduced affinity for both AFB1 and AFG1, respectively, and thus the differential recognition of AF26-T20-dU is only slightly better than AF26 (the ratio of *K*_d_ of AFG1 to AFB1 becomes ∼8-fold), suggesting that the hydrophobic interaction of methyl group of the T20 base has a slightly greater effect on the binding of aptamer to AFG1 than to AFB1.

We also attempted to replace the T20 with a C base, hypothesizing that the mutation may help the 4-NH_2_ groups of C form a hydrogen bond with the O4 atom of T14, thus facilitating the binding affinity to AFB1. However, for AFG1, the hydrogen bond may be weak or cannot form due to the steric effects, which could enlarge the difference between AFG1 and AFB1 in affinity to the aptamer. ITC results show that AF26-T20C improves the ratio of *K*_d_ of AFG1 to AFB1 to ∼17-fold, which is more than three times higher than that of AF26. When replaced with a 5mC base (AF26-T20-5mC), the recovery of hydrophobic interaction of the methyl group results in the ratio of *K*_d_ of AFG1 to AFB1 becoming ∼11-fold.

In summary, we utilize the high-resolution complex structure to elucidate the deeper mechanism of aptamer discriminatory recognition of AFB1 and AFG1, and precisely improve its ability to discriminate, providing a valuable example of structure-guided improvement of aptamer selectivity.

## DISCUSSION

### Base pair, triple and mismatch alignments on complex formation

The G19·C11 base pair and G6·G17·C13 triple are important structural components of the binding pocket. We speculated that disrupting their pairing would be directly detrimental to the binding affinity of AFB1. As expected, for the G19·C11 base pair and the G6·G17·C13 triple, ITC results show that any replacement of G base with A, T or C base, or the replacement of any C with A base, which destroys base pairing, causes the lost or dramatically decreased binding affinity (Supplementary Tables S2 and S7). The replacement of G17 or G19 base with inosine (AF26-G17I, AF26-G19I), which removes the hydrogen bond formed by the NH_2_–2 group of the G base, weakening base pairing, also reduces affinity by approximately an order of magnitude. However, replacing the G6 base with inosine (AF26-G6I) slightly enhances the binding affinity. We speculate that G6 in G6·G17·C13 triple may not be in the proper orientation, possibly due to the steric effect of the NH_2_–2 group (Supplementary Figure S10). Removing the NH_2_–2 group will facilitate the adjustment of G6 and thus stabilize the complex structure (although the hydrogen bond formed by the NH_2_–2 group is removed, a strong hydrogen bond may form between the H1 proton of G6 and N7 of G17).

The T5·T22 mismatch is also important in aptamer binding with AFB1, as T10 stacks with this T·T mismatch to help in the formation of complex structure and binding pocket. When the T5·T22 mismatch was substituted with canonical G·C and A·T Watson–Crick pairing, the aptamer affinity was reduced by ∼4-fold or more (AF26-T5A, *K*_d_ = 145 ± 15.4 nM; AF26-T22A, *K*_d_ = 103 ± 18 nM; AF26-C5G22, *K*_d_ = 158 ± 20.1 nM) (Supplementary Tables S2 and S7). These results suggest that T10 stacks better with T5·T22 than with canonical G·C and A·T base pairs, possibly due to the properties of bases. In addition, the substitution of T5 or T22 with dU does not cause large change in affinity (Supplementary Tables S2 and S7), showing that the methyl group of T5 and T22 does not significantly contribute in the interaction between T10 and T5·T22 mismatch.

### Stem length on complex formation

Maintaining a stable stem is crucial for preserving the overall structure and ensuring the aptamer can maintain a high affinity to AFB1. The importance of the stem with duplex structure was examined by analyzing the affinity of aptamers with varying length of stems through ITC (Supplementary Table S1). The shortest sequence that demonstrated efficient preservation of the high binding affinity to AFB1 is AF26, which contains a 5-bp stem with a T5·T22 mismatch. Longer stems (AF28, AF30 and AF32) do not improve affinity, but do improve the stability of the free aptamer. Free AF28, AF30 and AF32 aptamers can yield well-resolved signals of stem at 298 K (Supplementary Figure S11). The AF24 with a 3-bp stem shows a slight decrease in binding affinity (*K*_d_ = 50 ± 3.8 nM). However, any further truncation in the stem greatly destabilizes it and reduces the binding affinity, highlighting the critical role of a stable stem in maintaining the entire complex structure.

### The effect of temperature and metal ions on the binding affinity

The ITC experiments indicate that the binding is enthalpically favorable and entropically unfavorable, with negative enthalpy change (Δ*H*) and entropy change (Δ*S*) (Supplementary Figure S3 and Supplementary Table S9), suggesting that the binding process between the aptamer and AFB1 is enthalpically driven. The negative entropy change is a result of the aptamer transitioning from an unordered structure to a more rigid fold upon binding with AFB1, leading to an entropy loss. The van der Waals forces and hydrogen bonds are the main driving forces behind the interactions between aptamer and AFB1, causing heat release. Furthermore, it was also observed that the binding affinity of AF26 to AFB1 is dependent on temperature, with a stronger binding observed at lower temperature (Supplementary Table S9). The temperature-sensitive binding affinity of AF26 is likely because the lower temperature favors the stabilization of the stem of free aptamer and facilitates the binding of ligand. At low temperatures, the free aptamer has a more stable stem in the absence of AFB1 (Figure 1), resulting in smaller changes in Δ*H* and Δ*S* during the aptamer’s binding, as the aptamer undergoes a smaller structural change due to fewer base pair formation. At high temperature, the stem of the free aptamer is more unstable, and AFB1 binding induces the aptamer to fold into an ordered structure with more base pair formation and heat release, resulting in larger Δ*H* and Δ*S* values. With increasing temperature, Δ*H* and Δ*S* values increased, indicating that the binding-induced structural change of the aptamer becomes larger with temperature as the stem structure of free aptamer is less ordered in the absence of AFB1.

The affinity analyses also indicate that Mg^2+^ ions are favorable and efficient for AFB1 binding to the aptamer (Supplementary Table S10). Without Mg^2+^ in the buffer, the aptamer’s affinity is greatly reduced by over 40-fold (*K*_d_ = 1180 ± 303 nM). It is known that Mg^2+^ ions can facilitate base pairing in DNA duplex structure (52). To further understand how Mg^2+^ ions stabilize the aptamer complex structure and facilitate the AFB1 binding, we performed Mg^2+^ titration experiments on the imino protons of AF26 aptamer in the presence of AFB1 at 278 K (Supplementary Figure S12). The experimental results revealed that the resonances of imino protons of G6 in the bulged loop and the T5 and T22 from the stem shifted obviously as the concentration of Mg^2+^ increased. From the AFB1–AF26 complex structure, it is clear that the G6, T5 and T22 are located close to the G9–C11 region, which is the valley transiting from the G6–T10 bugle to the hairpin structure. This is reflected by the distances between the negatively charged phosphate groups, as shown in Supplementary Figure S13. The accumulation of negative charges in these regions may need Mg^2+^ to stabilize the structure of the complex. In addition, Mg^2+^ helps to enhance base pairing and stem stability, which is important for the aptamer affinity. Accordingly, stem length, temperature and Mg^2+^ all significantly affect the stability of the aptamer and its affinity.

## CONCLUSION

In conclusion, we determined the high-resolution structure of AFB1–AF26 DNA aptamer by solution NMR spectroscopy, which presents a new remarkable example of an adaptive conformational transition that allows the loop of the aptamer to create a unique binding pocket. The AF26 aptamer forms its AFB1 binding pocket through an induced-fit mechanism, by transiting its unstructured loop region into the well-defined bulged loops and hairpin structures. The hairpin folds toward the bulged loop by base pairing and hydrophobic interactions, almost entirely enclosing AFB1. Furthermore, based on the complex structures, we uncover the molecular mechanism of the aptamer’s discriminatory recognition of AFB1 and AFG1, and its discriminatory ability was even improved by introducing a single base mutation. Our work contributes to a better understanding of aptamer’s high-affinity recognition mechanism, and also serves as an important foundation for the design and optimization of aptamers and aptamer-based biosensors and applications.

## Supplementary Material

gkad541_Supplemental_File

## Data Availability

The NMR assignments and structures of the AFB1–AF26 complexes have been deposited in the Biological Magnetic Resonance Data Bank (ID: 36549) and Protein Data Bank (ID: 8IF5).
