# Supplementary material for: Structural basis for high-affinity recognition of aflatoxin B1 by a DNA aptamer
Source: Nucleic Acids Res. 2023 Jun 23;51(14):7666–74. doi: 10.1093/nar/gkad541 (PMC10415127; doi:10.1093/nar/gkad541)
Supplement: gkad541_Supplemental_File [file gkad541_supplemental_file.pdf]

Supplemental Information

Structural Basis for High-Affinity Recognition of Aflatoxin B1 by a DNA Aptamer

Guohua Xu[1, +], Chen Wang[1, 4, +], Hao Yu[2, 4, +], Yapiao Li[2, 4], Qiang Zhao[2, 3, 4, *], Xin Zhou[1], Conggang Li[1, *] and Maili Liu[1]

[1] Key Laboratory of Magnetic Resonance in Biological Systems, State Key Laboratory of Magnetic Resonance and Atomic and Molecular Physics, National Center for Magnetic Resonance in Wuhan, Wuhan National Laboratory for Optoelectronics, Wuhan Institute of Physics and Mathematics, Innovation Academy for Precision Measurement Science and Technology, Chinese Academy of Sciences, Wuhan, 430071, P. R. China.

[2] State Key Laboratory of Environmental Chemistry and Ecotoxicology, Research Center for Eco-Environmental Sciences, Chinese Academy of Sciences, Beijing, 100085, P. R. China.

[3] School of Environment, Hangzhou Institute for Advanced Study, UCAS, Hangzhou, 310024, P. R. China.

[4] University of Chinese Academy of Sciences, Beijing, 100049, P.R. China.

[+] These authors contributed equally to this work.

* To whom correspondence should be addressed. Email: conggangli@wipm.ac.cn (Conggang Li) qiangzhao@rcees.ac.cn (Qiang Zhao)

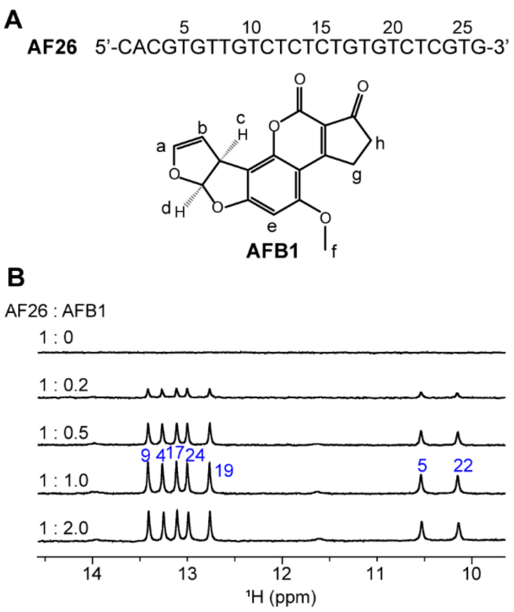

Figure S1. ^{1}H NMR spectra of aptamer. (A) Sequence of aptamer AF26 and chemical structure of AFB1. (B) Imino regions of ^{1}H NMR spectra of aptamer AF26 titrated with AFB1 at 298 K. The signal assignments of imino protons from bound aptamers were marked in blue fonts.

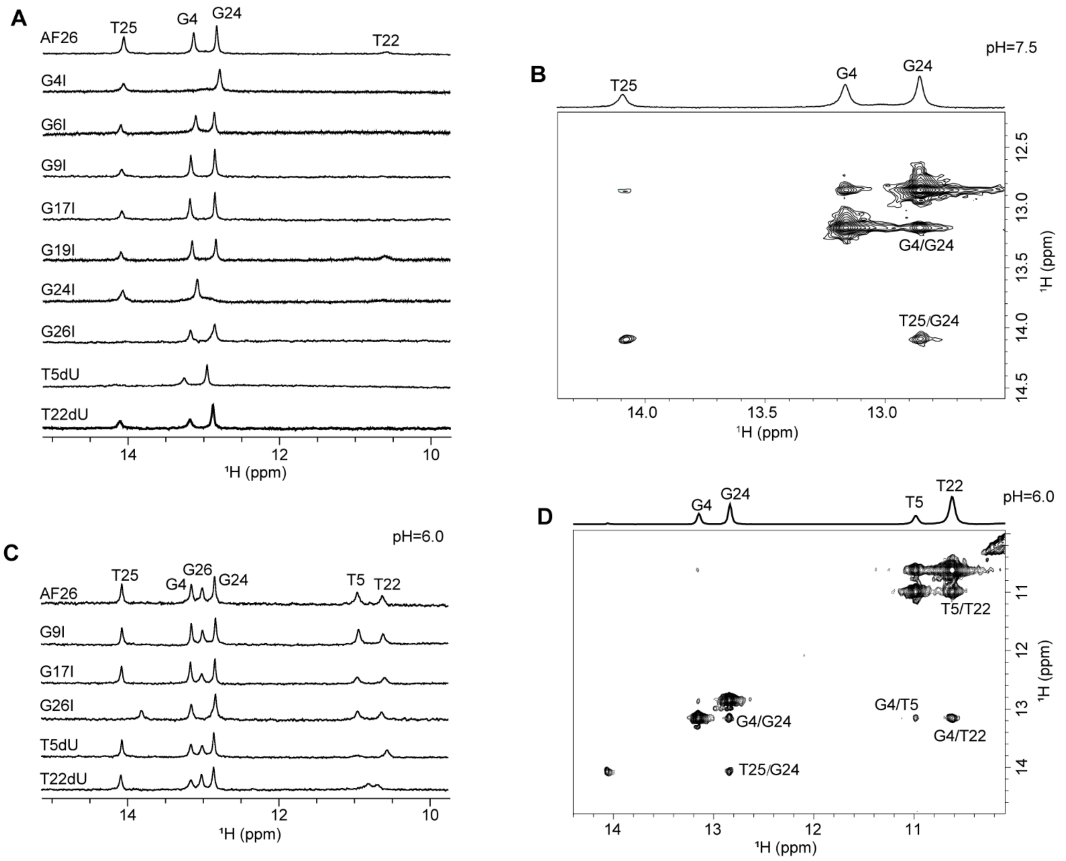

Figure S2. (A and C) Imino regions of ^{1}H NMR spectra of free AF26 aptamer with G-to-I and T-to-dU substitution at pH 7.5 and 6.0, respectively. (B and D) NOESY spectrum (mixing time, 120 ms) of free AF26 in H_2O buffer at pH 7.5 and 6.0, respectively, showing the connectivities between the imino protons. All experiments were acquired at 278 K.

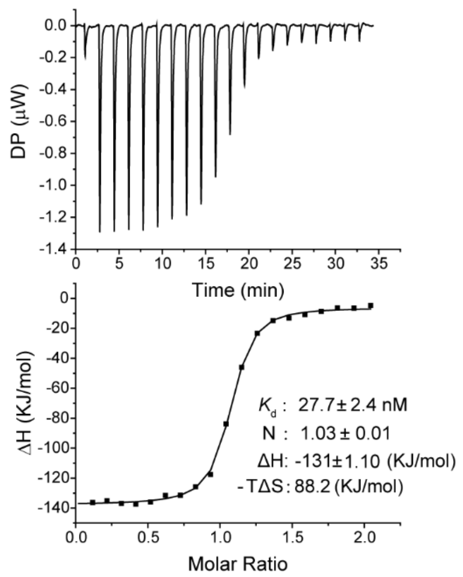

Figure S3. The ITC titration curves for the binding of AF26 aptamer and AFB1 at 298 K.

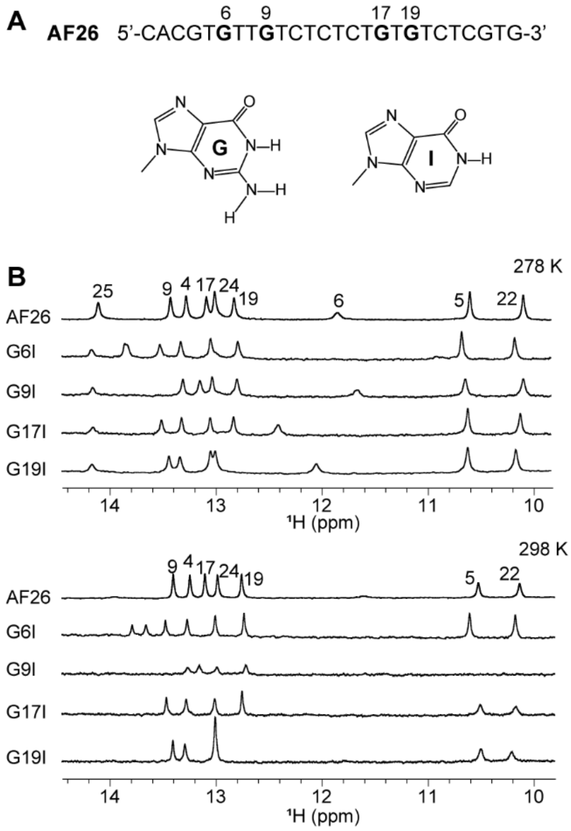

Figure S4. ^{1}H NMR spectra of AF26 aptamer and its mutants. (A) Sequence of aptamer AF26 and chemical structure of G and I bases. (B) Imino regions of ^{1}H NMR spectra of AF26 aptamer with the replacement of G by I in the presence of AFB1 at 278 K and 298 K.

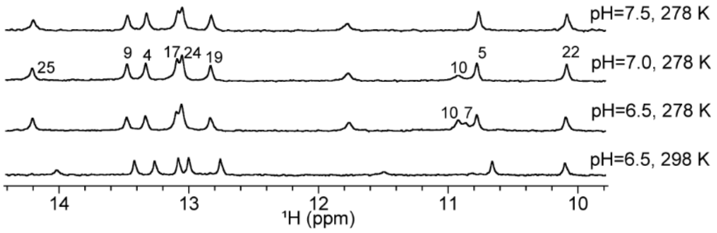

Figure S5. Imino regions of ¹H NMR spectra of the AF26-AFB1 complex at different pH.

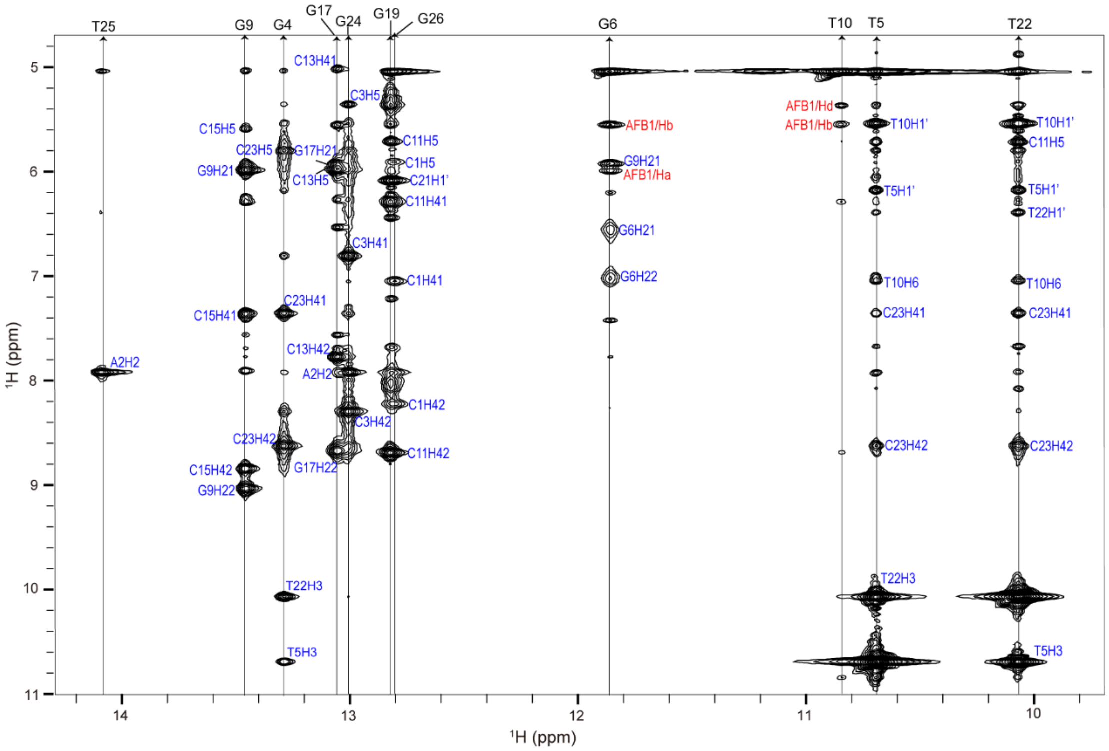

Figure S6. Expanded NOESY spectrum (mixing time, 120 ms) of AF26-AFB1 complex in H_2O buffer at 278 K, showing the connectivities between the imino and the amino/base protons. The intermolecular cross peaks between AFB1 and AF26 protons were marked in red fonts.

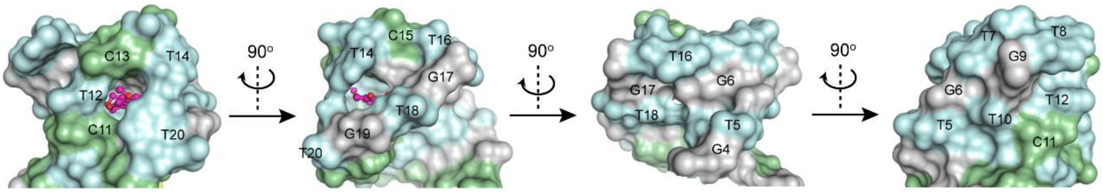

Figure S7. The surface view of AFB1 (stick) in binding cavity of aptamer AF26 showing that the AFB1 was tightly wrapped by binding pockets.

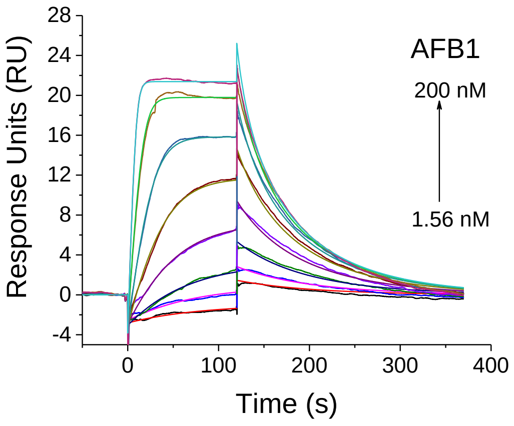

Figure S8. Sensorgrams of AFB1 at different concentrations ranging from 1.56 nM to 200 nM AFB1 in SPR analysis. Kinetic Analysis: k_{on}=2.11 $\times10^{6}$ $M^{-1}S^{-1}$, k_{off}=0.046 S^{-1} ; K_d=21.8 nM.

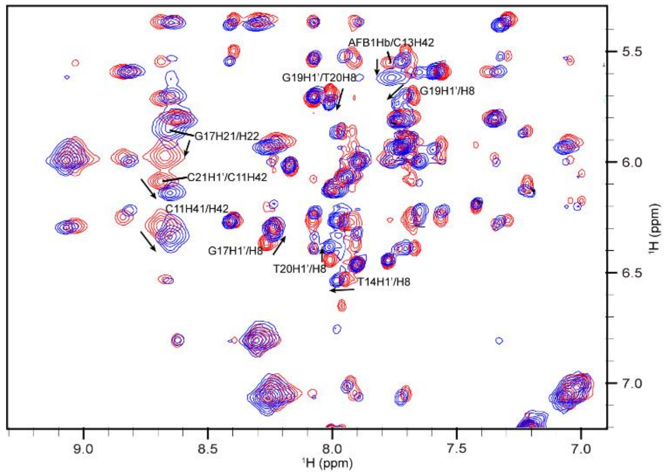

Figure S9. The overlap of 2D NOESY spectra of AF26-AFB1 (red) and AF26-AFG1 (blue) complexes in H_2O buffer at 278 K. The obviously moved peaks are labeled in the figure.

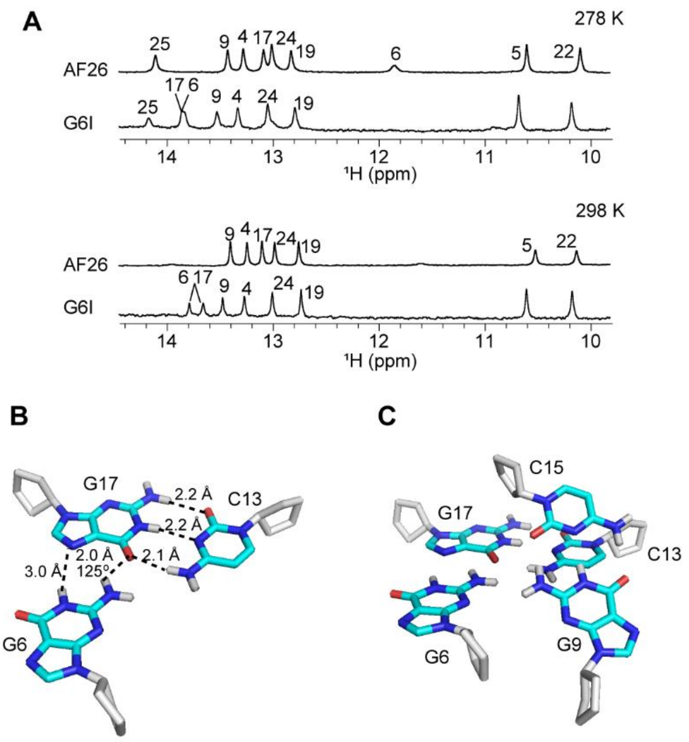

Figure S10. (A) The comparison of ^{1}H NMR spectra between AF26 and AF26-G6I mutant aptamers in the presence of AFB1 at 278 K and 298 K. (B)Hydrogen-bonding alignments of G6·G17·C13 triple. (C) The alignments of G6·G17·C13 triple and G9·C15 base pair.

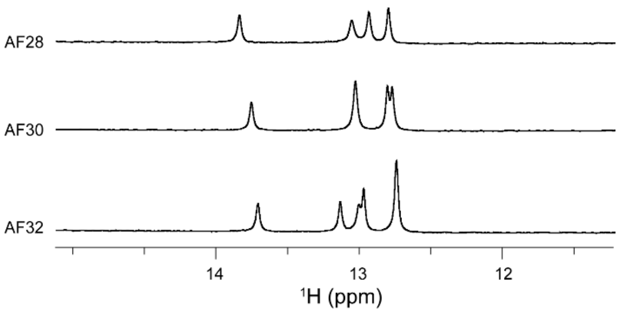

Figure S11. Imino regions of ^{1}H NMR spectra of free AF28, AF30 and AF32 aptamers at 298 K.

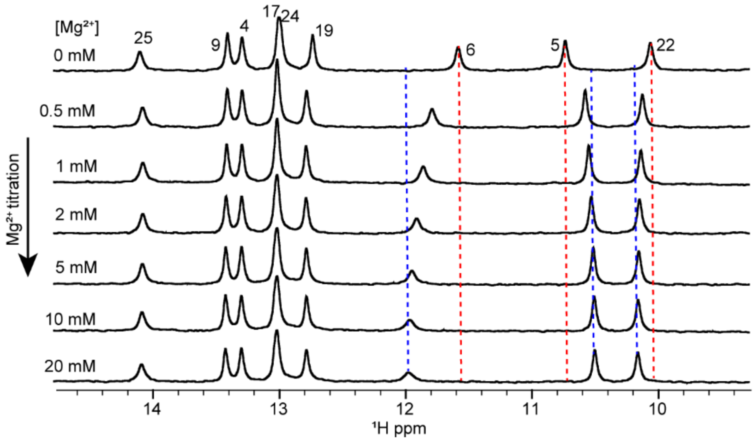

Figure S12. NMR titration experiments showing the effect of Mg^{2+} on the imino proton region of AF26 aptamer in the presence of 2 equivalent AFB1. NMR spectra were acquired in 10 mM Tris (pH 7.5) buffer at 278 K.

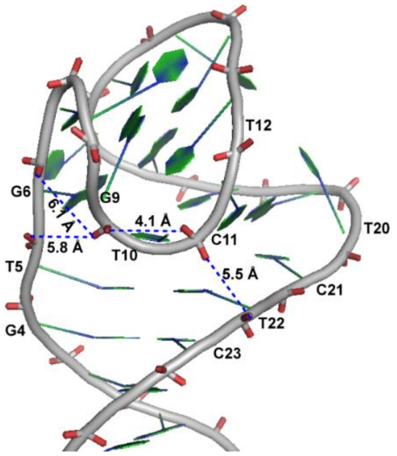

Figure S13. The mean structure of AF26-AFB1 complex showing the distances between negatively charged phosphate groups.

Table S1. Sequence and dissociation constant of aptamers determined by ITC at 298 K.

Name	Sequence (5' to 3')	K_d /nM	ΔH (KJ/mol)	-TΔS (KJ/mol)
A32	GGG CAC GTG TTG TCT CTC TGT GTC TCG TGC CC	30.1±3.2	-124 ±1.44	81.2
A30	GGC ACG TGT TGT CTC TCT GTG TCT CGT GCC	29.3±2.8	-119 ±1.24	76.1
A28	GCA CGT GTT GTC TCT CTG TGTCTC GTG C	30.9±2.3	-123 ±1.16	79.5
A26	CAC GTG TTG TCT CTC TGT GTC TCG TG	27.7±2.4	-131 ±1.10	88.2
A24	ACG TGT TGT CTC TCT GTG TCT CGT	50±3.8	-161 ±1.59	118.8
A22	CGT GTT GTC TCT CTG TGT CTC G	341±20	-171 ±2.63	133.9
A20	GTG TTG TCT CTC TGT GTC TC	2520±839	-137 ±27.0	104.6
A18	TG TTG TCT CTC TGT GTC T	1430±336	-132 ±13.5	98.7
A16	G TTG TCT CTC TGT GTC	21900±189000	-335 ±5272	307.9

Table S2. AF26 aptamer and its variants sequences.

**Name**	**Sequence (5’to 3’)**
AF26	CAC GTG TTG TCT CTC TGT GTC TCG TG
AF26-G4I	CAC **I**TG TTG TCT CTC TGT GTC TCG TG
AF26-T5A	CAC G**A**G TTG TCT CTC TGT GTC TCG TG
AF26-T5-dU	CAC G**(dU)**G TTG TCT CTC TGT GTC TCG TG
AF26-G6I	CAC GT**I** TTG TCT CTC TGT GTC TCG TG
AF26-G6T	CAC GT**T** TTG TCT CTC TGT GTC TCG TG
AF26-G6A	CAC GT**A** TTG TCT CTC TGT GTC TCG TG
AF26-G6C	CAC GT**C** TTG TCT CTC TGT GTC TCG TG
AF26-T7-dU	CAC GTG **(dU)**TG TCT CTC TGT GTC TCG TG
AF26-G9I	CAC GTG TT**I** TCT CTC TGT GTC TCG TG
AF26-G9T	CAC GTG TT**T** TCT CTC TGT GTC TCG TG
AF26-G9A	CAC GTG TT**A** TCT CTC TGT GTC TCG TG
AF26-G9C	CAC GTG TT**C** TCT CTC TGT GTC TCG TG
AF26-T10A	CAC GTG TTG **A**CT CTC TGT GTC TCG TG
AF26-T10-dU	CAC GTG TTG **(dU)**CT CTC TGT GTC TCG TG
AF26-T12A	CAC GTG TTG TC**A** CTC TGT GTC TCG TG
AF26-T12-dU	CAC GTG TTG TC**(dU)** CTC TGT GTC TCG TG
AF26-T14A	CAC GTG TTG TCT C**A**C TGT GTC TCG TG
AF26-T14-dU	CAC GTG TTG TCT C**(dU)**C TGT GTC TCG TG
AF26-T16-dU	CAC GTG TTG TCT CTC **(dU)**GT GTC TCG TG
AF26-G17I	CAC GTG TTG TCT CTC T**I**T GTC TCG TG
AF26-G17T	CAC GTG TTG TCT CTC T**T**T GTC TCG TG
AF26-G17A	CAC GTG TTG TCT CTC T**A**T GTC TCG TG
AF26-G17C	CAC GTG TTG TCT CTC T**C**T GTC TCG TG
AF26-T18A	CAC GTG TTG TCT CTC TG**A** GTC TCG TG
AF26-T18-dU	CAC GTG TTG TCT CTC TG**(dU)** GTC TCG TG
AF26-G19I	CAC GTG TTG TCT CTC TGT **I**TC TCG TG
AF26-G19T	CAC GTG TTG TCT CTC TGT **T**TC TCG TG
AF26-G19A	CAC GTG TTG TCT CTC TGT **A**TC TCG TG
AF26-G19C	CAC GTG TTG TCT CTC TGT **C**TC TCG TG
AF26-T20A	CAC GTG TTG TCT CTC TGT GCC **A**CG TG
AF26-T20C	CAC GTG TTG TCT CTC TGT G**C**C TCG TG
AF26-T20-5mC	CAC GTG TTG TCT CTC TGT G**(5mC)**C TCG TG
AF26-T20-dU	CAC GTG TTG TCT CTC TGT G **(dU)**C TCG TG
AF26-G24I	CAC GTG TTG TCT CTC TGT GTC TC**I** TG
AF26-T22A	CAC GTG TTG TCT CTC TGT GTC **A**CG TG
AF26-T22-dU	CAC GTG TTG TCT CTC TGT GTC **(dU)**CG TG
AF26-C5G22	CAC G**C**G TTG TCT CTC TGT GTC **G**CG TG
AF26-G26I	CAC GTG TTG TCT CTC TGT GTC TCI T**I**

Table S3. Proton chemical shift of AF26 in the AF26-AFB1 binary complex. The chemical shift values for carbon hydrogens and active hydrogens are from NMR spectra acquired in D_2O buffer (pH 7.5) at 278 K and from NMR spectra acquired in H_2O buffer (pH 7.5) at 278 K, respectively.

Residue	H1/H3	H41/H21 /H61	H42/H22 /H62	H5/Me /H2	H1'	H2'	H2"	H3'	H4'	H5'	H5"	H8/H6
C1		7.05	8.22	5.90	5.51	1.93	2.37	4.70	4.06	3.77	3.77	7.70
A2				7.92	6.27	2.80	2.93	5.04	4.44	4.14	4.01	8.40
C3		6.81	8.30	5.35	5.52	1.83	2.17	4.83	4.13	4.26	4.00	7.30
G4	13.29				6.05	2.72	2.64	5.04	4.42	4.12	4.00	7.93
T5	10.69			1.72	6.18	1.73	2.83	5.06	4.09			6.99
G6	11.86	6.56	7.02		6.29	3.11	2.70	5.16	5.25	4.09	4.36	8.24
T7				1.87	5.79	1.80	2.25	4.61	3.33	3.66	3.72	7.35
T8				1.88	6.44	2.29	2.48	4.53	4.53	4.08	3.99	7.78
G9	13.46	5.98	9.03		6.02	2.85	2.85	5.06	4.53	3.97	4.19	8.17
T10	10.84			0.66	5.54	1.67	1.62	4.87	4.26	3.98	3.98	7.04
C11		6.29	8.69	5.71	6.26	1.12	2.28	4.70	4.38	4.10	3.93	8.08
T12	7.90			1.68	6.26	2.61	2.47	5.05	4.23	4.07	4.02	7.57
C13		5.03	7.77	5.96	6.27	2.12	2.62	5.08	4.54	4.16	4.22	7.68
T14				2.08	6.52	2.18	2.29	4.83	4.44	4.27	4.19	7.96
C15		7.36	8.84	5.58	5.92	1.91	2.35	4.64	3.47	4.07	4.02	7.56
T16				1.62	6.20	1.99	2.44	4.70	4.39	3.33	2.80	7.43
G17	13.05	5.97	8.67		6.36	2.98	2.81	5.11	4.36	4.22	4.19	8.27
T18				2.01	6.46	2.37	2.66	4.99	4.53	4.30	4.21	7.91
G19	12.82				5.69	2.54	2.28	5.04	4.45	4.25	4.14	7.68
T20				2.06	6.44	1.96	2.17	4.69	4.39	4.27	4.12	8.02
C21				5.21	6.08	1.70	2.34	4.61	3.65	3.81	3.99	7.22
T22	10.07			1.90	6.39	1.83	1.46	4.75	4.12	3.85	3.39	7.68
C23		7.36	8.62	5.79	4.99	2.46	2.40	4.89	4.28	4.06	4.01	7.73
G24	13.01				6.11	2.68	2.82	5.04	4.44	4.15	4.15	8.01
T25	14.09			1.55	5.86	1.94	2.35	4.89	4.19	4.26	4.19	7.22
G26	12.81				6.08	2.69	2.42	4.72	4.22	4.11	4.15	7.96

Table S4. Proton chemical shifts of bound AFB1 and AFG1 in the AF26-AFB1/AFG1 binary complexes.

Protons	δ_H (AFB1)	δ_H (AFG1)
Ha(CH)	5.99	6.01
Hb(CH)	5.55	5.62
Hc(CH)	4.31	4.39
Hd(CH)	5.37	5.38
He(CH)	6.08	6.10
Hf(CH_3)	3.73	3.73
Hg(CH_2)	2.55	3.90
Hh(CH_2)	2.07, 2.29	4.05

Table S5. Unambiguously assigned intermolecular NOEs between AFB1/AFG1 and AF26 aptamer protons in AF26-AFB1 complex.

**AFB1/AFG1 protons**	**AF26 protons in AF26-AFB1 complex**	**AF26 protons in AF26-AFG1 complex**
Ha	T10-H6, Me<br>G6-H1<br>T12-H3	T10-H6, Me<br>G6-H1<br>T12-H3
Hb	T10-Me, H3<br>G6-H1<br>T12-H3	T10-Me, H3<br>G6-H1<br>T12-H3
Hc	T10-H3<br>G19-H1<br>C11-H42	T10-H3<br>G19-H1<br>C11-H42
Hd	T10-H3, H6, H1’, H2’/2’’, H3’<br>C11-H5, H6, H42<br>T12-H3<br>G19-H1	T10-H3, H6, H1’, H2’/2’’, H3’<br>C11-H5, H6, H42<br>T12-H3<br>G19-H1
He	T12-H6, H1’, H4’, H5’/5’’<br>C13-H5, H6<br>C11-H6, H2’/2’’, H3’	T12-H1’, H4’, H5’/5’’<br>C13-H5, H6<br>C11-H6, H2’/2’’, H3’
Hf	C13-H5, H6, H4’, H5’<br>T12-H1’, H3’, H4’, H5’/5’’<br>G19-H1<br>C11-H2’/2’’	C13-H5, H6, H4’, H5’<br>T12-H6, H1’, H3’, H4’, H5’/5’’<br>G19-H1<br>C11-H2’/2’’
Hg	T14-Me<br>T20-Me	T14-Me<br>C13-H1’<br>T20-Me, H6<br>G19-H1’, H8
Hh	T14-Me<br>T20-Me	T14-Me<br>T20-Me, H6<br>G19-H1’, H8

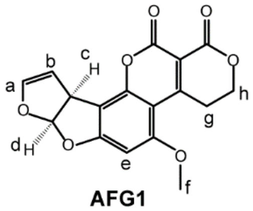

AFG1

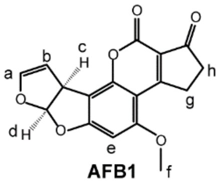

AFB1

Table S6. Statistics of the computed ten structures of AF26-AFB1 and AF26-AFG1 complexes

	**AF26-AFB1**	**AF26-AFG1**
**Distance restraints**		
Intraresidue	80	80
Sequencial	147	147
Long-range	119	119
Intermolecular	49	53
**Other restraints**		
Hydrogen bond restraints	61	61
Sugar pucker restraints	52	52
Dihedral angles	68	68
**Repulsive restraints**	4	4
**NOE violations**		
Number (>0.2 Å)	0	0
RMSD of vilations (Å)	0.021 ± 0.002	0.022 ± 0.002
**Deviations from the ideal covalent geometry**		
Bond lengths (Å)	0.002 ± 0.000	0.002 ± 0.000
Bond angles (deg)	0.417 ± 0.006	0.419 ± 0.008
Impropers (deg)	0.293 ± 0.006	0.320 ± 0.003
**Pairwise all heavy atoms RMSD values (Å)**		
Entire complex	0.54 ± 0.17	0.75 ± 0.28
Entire complex less ligands	0.55 ± 0.17	0.76 ± 0.28

Table S7. The dissociation constant of AF26 aptamer and its variants determined by ITC.

**Aptamer**	K_d **/ nM**	**ΔH (KJ/mol)**	**-TΔS (KJ /mol)**
AF26	27.7±2.4	-131 ±1.10	88.2
AF26-T5A	145±15.4	-104±2.18	64.9
AF26-T5-dU	34.8±4.0	-134 ±1.68	91.6
AF26-G6I	19.8 ±3.25	-150 ±2.19	106
AF26-G6C	NB		
AF26-G6A	NB		
AF26-G6T	2880±5460	-39.5±39.9	7.87
AF26-T7A	NB		
AF26-T7-dU	69.4±10.9	-126 ±2.87	85.3
AF26-G9T	NB		
AF26-G9A	NB		
AF26-G9C	NB		
AF26-G9I	NB		
AF26-T10A	NB		
AF26-T10-dU	248±41	-124 ±4.55	86.0
AF26-T12A	NB		
AF26-T12-dU	68.8±13.4	-135 ±3.35	93.7
AF26-T14A	1530±511	-122±16.8	88.7
AF26-T14-dU	58.0±6.0	-136 ±1.72	95.1
AF26-T16A	2840±2190	-118±44.8	86.2
AF26-T16-dU	101 ±15.8	-126 ±3.09	86.0
AF26-G17I	401 ±41.0	-151 ±3.8	114
AF26-G17C	NB		
AF26-G17T	NB		
AF26-G17A	NB		
AF26-T18A	2000±1500	-52.3±17.8	19.9
AF26-T18-dU	30.5 ±5.4	-125 ±2.33	82.0
AF26-G19I	345 ±40.1	-134 ±3.64	97.0
AF26-G19T	2000±1500	-44.8±105	14.5
AF26-G19A	NB		
AF26-G19C	NB		
AF26-T20A	3280±3780	-64.0±37.0	32.9
AF26-T20-dU	46.0±5.5	-120 ±1.68	77.7
AF26-T22A	103±18	-163±4.16	123.0
AF26-T22-dU	38.4 ±8.1	-135 ±3.09	93.1
AF26-C5G22	158±20.1	-131±2.85	92.0

NB: no binding.

Table S8. The dissociation constant of AF26 aptamer and its variants determined by ITC.

Aptamer	K_d for AFB1/ nM	K_d for AFG1/ nM	K_d (AFG1)/ K_d (AFB1)
AF26	27.7±2.4	144±26.8	5.2
AF26-T20-dU	46.0±5.5	367±42.6	8
AF26-T20C	19.1±3.1	333±54.8	17.4
AF26-T20-5mC	16.1±2.5	174±27.1	10.8

Table S9. The dissociation constant of AF26 aptamer determined by ITC at various temperatures, showing the effect of temperature on the dissociation constant.

Temperature /℃	K_d / nM	ΔH (KJ/mol)	-TΔS (KJ /mol)	ΔS (KJ /mol)
5	4.3 ±1.9	-74.8 ±1.58	30.2	-0.109
10	5.7 ±2.0	-91.3 ±1.95	46.5	-0.164
15	12.6 ±2.7	-101 ±1.7	57.6	-0.200
20	17.2 ±3.3	-111 ±2.33	67.7	-0.231
25	27.7 ±2.4	-131 ±1.10	88.2	-0.296
30	56.5 ±5.0	-153 ±1.87	111	-0.366
35	162 ±15.8	-186 ±3.27	145	-0.471

Table S10. The dissociation constant of AF26 aptamer determined by ITC at various $MgCl_2$ concentration, showing the effect of $MgCl_2$ concentration on the dissociation constant.

$MgCl_2$ concentration / mM	K_d / nM	ΔH (KJ/mol)	-TΔS (KJ/mol)
0	1180±303	-210 ±20.4	176
2	42.0±8.6	-141 ±3.22	99.2
10	28.7±6.2	-137 ±2.85	94.3
50	24.2±5.1	-140 ±2.65	96.8

Binding buffer: 10 mM Tris-HCl (7.5), 2% DMSO, 0.1% Tween20, at 25 ºC
